# Supplementary material for: In Vivo Detection of Circulating Cancer-Associated Fibroblasts in Breast Tumor Mouse Xenograft: Impact of Tumor Stroma and Chemotherapy
Source: Cancers (Basel). 2023 Feb 9;15(4):1127. doi: 10.3390/cancers15041127 (PMC9954095; doi:10.3390/cancers15041127)
Supplement: Supplementary file 1 [file cancers-15-01127-s001.zip › cancers-2100724-supplementary.pdf]

Supplementary data

Detection of circulating cancer-associated fibroblasts in vivo in breast tumor mouse xenograft: impact of tumor stroma and chemotherapy

Tao Lu[a], Lisa Oomens[b], Leon W.M.M. Terstappen[c], Jai Prakash[a]

[a]Engineered Therapeutics, Department of Advanced Organ bioengineering and Therapeutics, TechMed Centre, Faculty of Science and Technology, University of Twente, Drienerlolaan 5, 7500AE, Enschede, the Netherlands

[b]VyCAP B.V., Capitool 41, 7521 PL Enschede, The Netherlands

[c]Medical Cell BioPhysics, Faculty of Science and Technology, University of Twente, Hallenweg 23, 7522 NH Enschede, The Netherlands

*Corresponding author: j.prakash@utwente.nl

Supplementary data

Supplementary table S1. Antibody used and corresponding concentration.

Antibody	Dilution/Concentration used in imaging
FAP Alexa-647 conjugated antibody	1:60 / 3 μg/ml
ITGA 5 Alexa-488 conjugated antibody	1:100 / 2 μg/ml
α-SMA Alexa-488 conjugated antibody	1:500 / 1 μg/ml
pan-cytokeratin PE-conjugated antibody	1: 100 / 1 μg/ml
pan-cytokeratin eFluor 570 conjugated antibody	1:100 / 2 μg/ml
vimentin Alexa-488 conjugated antibody	1:10 / 2 μg/ml
Hoechst	1:10000 / 1 μg/ml
α-SMA unconjugated antibody (for Western plot)	1:1000

Supplementary data

Supplementary figures

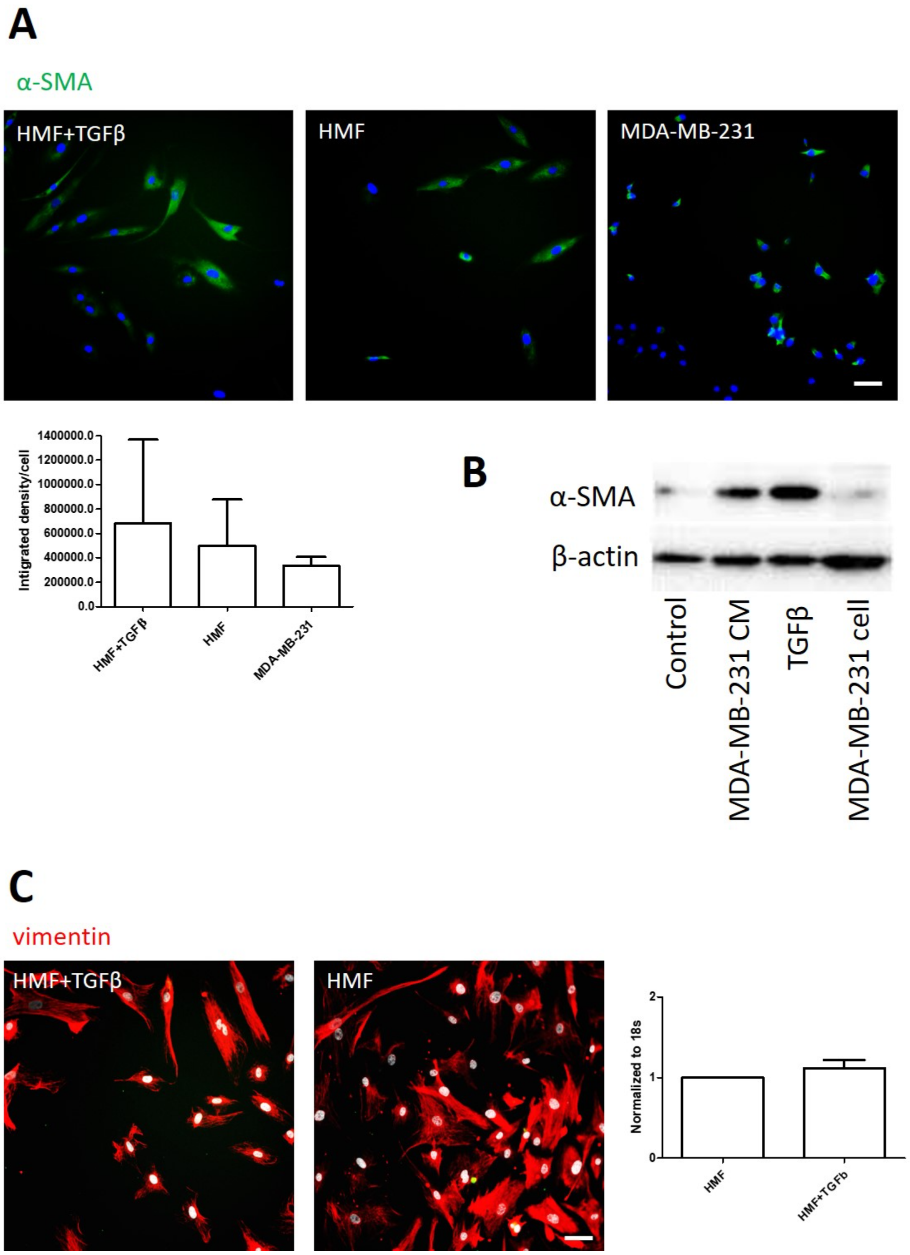

Supplementary figure S1. *(A) Immunofluorescence staining for α-SMA (green) in non-activated and TGFβ-activated HMFs as well as MDA-MB-231 breast tumor cells, while Hoechst (blue) represents nuclear staining. Not significant difference from staining was observed. Scale bar, 100 µm. (B) Western blot assay showed the increased protein expression of α-SMA in HMFs activated by TGFβ or cancer cell conditioned medium. (C) TGFβ-activated and non-activated and HMFs did not show immunofluorescence staining for vimentin (red), as well as for the gene expression. Scale bar, 100 µm.*

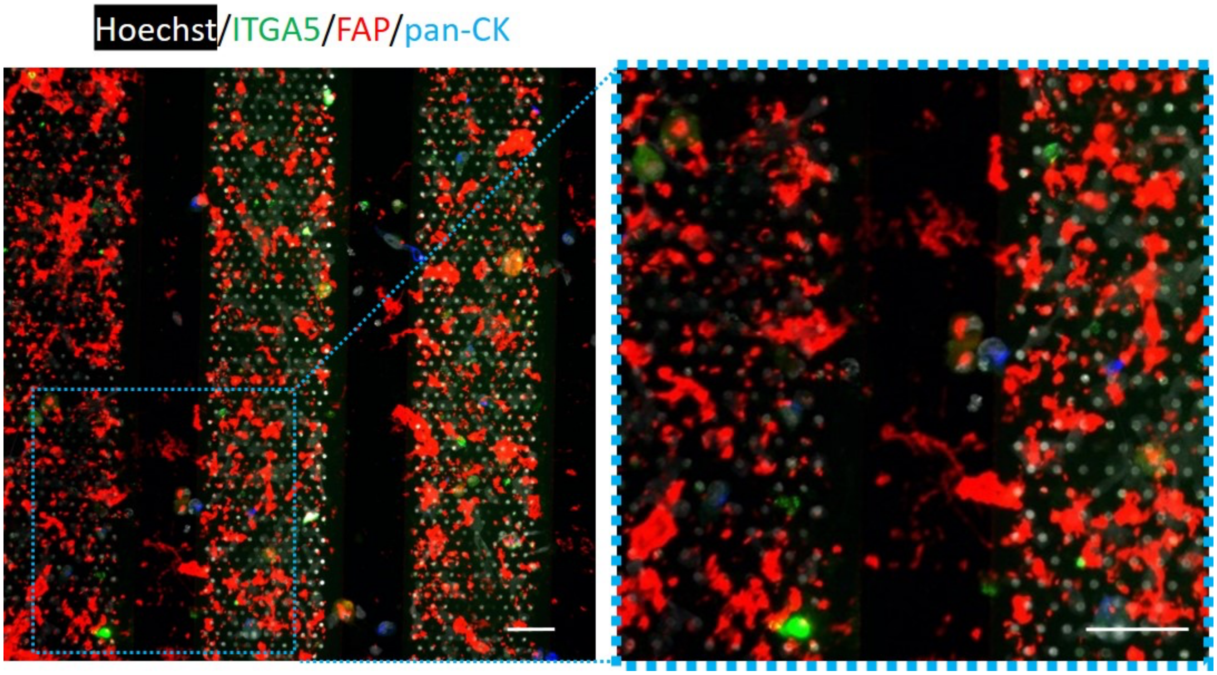

Supplementary figure S2. *Immunofluorescence staining for ITGA5 (green), FAP (red) and pan-CK (blue) in fresh mouse blood mixed with HMFs and MDA-MB-231 breast tumor cells, while Hoechst (white) represents nuclear staining. Strong unspecific binding of FAP in blood was shown. Scale bar, 40 μm.*

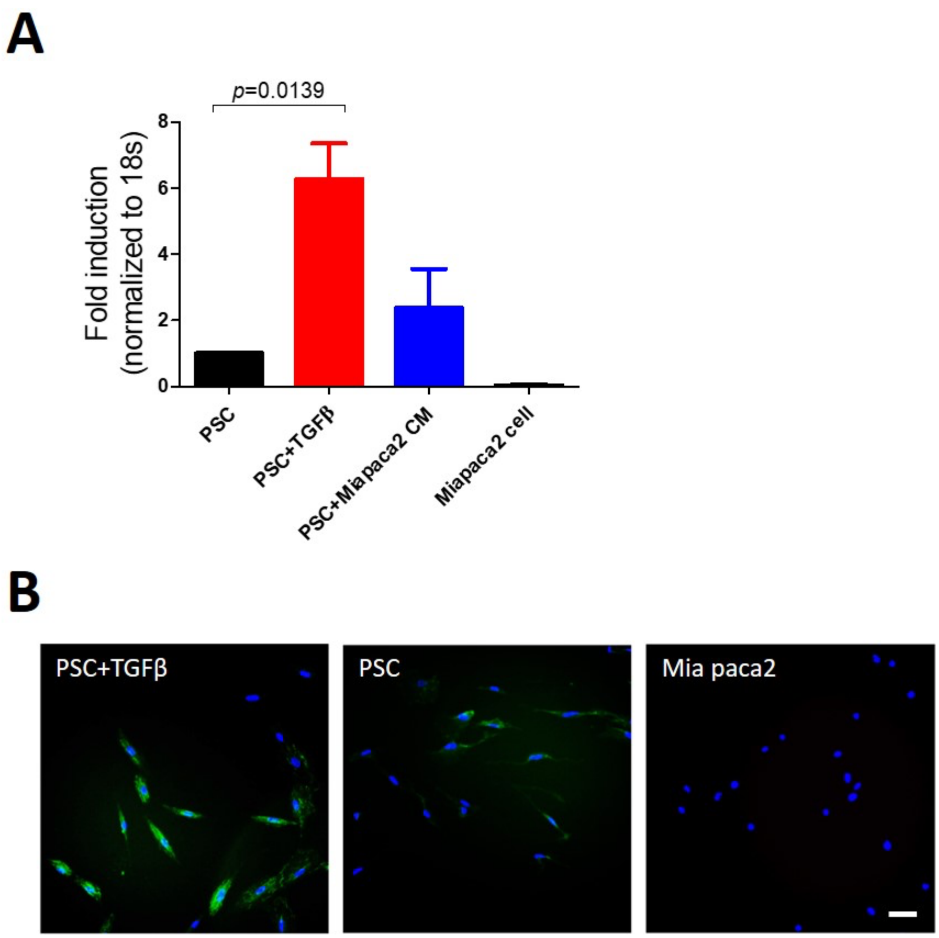

Supplementary figure S3. *(A) Gene expression of ITGA5 in pancreatic stellate cells (PSCs) with non-activation or activation with TGFβ or conditional medium collected from pancreatic cancer cells, and Mia paca2 pancreatic cancer cells. (B) Immunofluorescence staining for ITGA5 (green) in non-activated and TGFβ-activated PSCs as well as MIA PaCa2 pancreatic cancer cells, while Hoechst (blue) represents nuclear staining. Scale bar, 100 µm.*

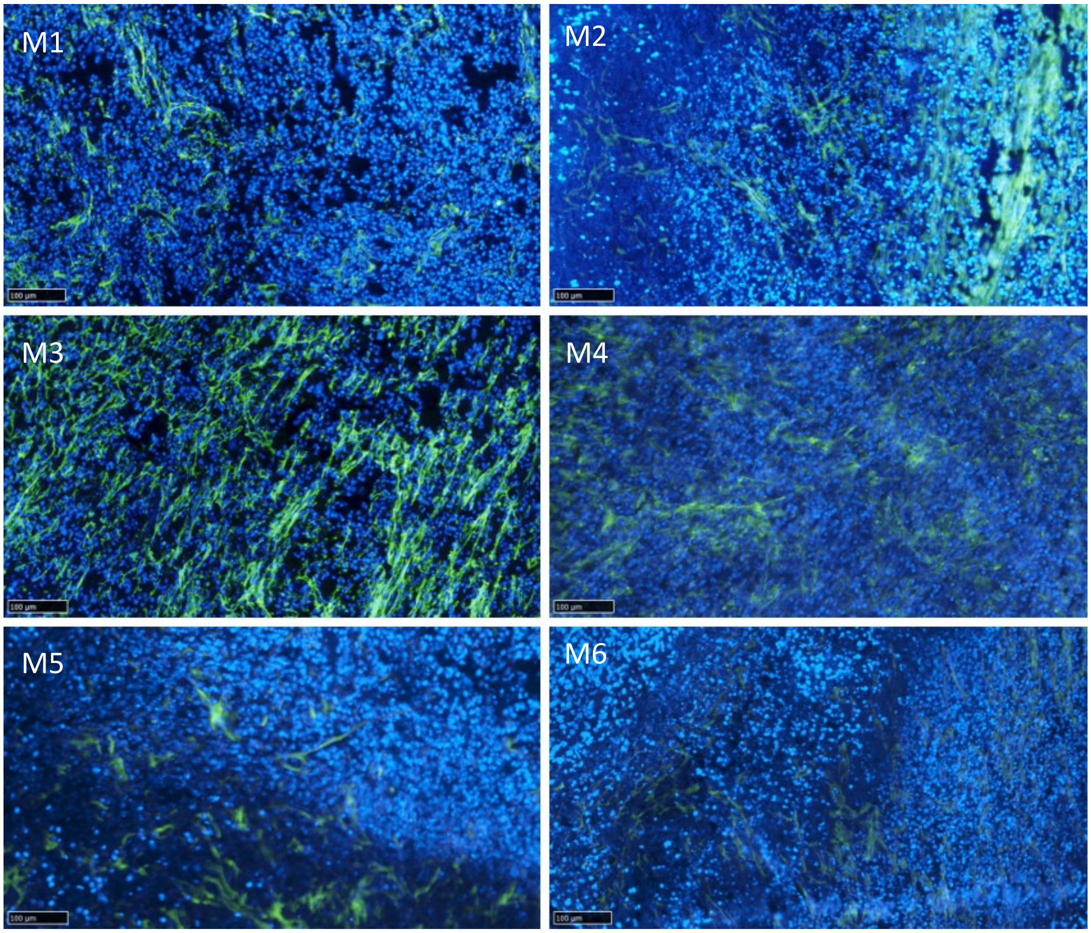

Supplementary figure S4. *Immunofluorescence staining for collagen (green), in 6 mice implanted with tumor cells only. All mice showed collagen positive areas in their tumor slices, indicating the existence of CAFs which might be from EMT of tumor cells or recruitment of host fibroblasts. Scale bar, 100 μm.*
